# Combined Aerobic and Resistance Training Improves Metabolic Health and Is Associated with Arginine and Histidine Metabolic Changes During the Transition from Metabolically Unhealthy to Metabolically Healthy Obesity in Young Adults

**DOI:** 10.3390/nu18121956

**Published:** 2026-06-17

**Authors:** Xueyin Fei, Min Wu, Yanchun Li, Mengru Zhang, Xiangang Yang

**Affiliations:** 1 Department of Exercise Biochemistry, Sport Science School, Beijing Sport University, Beijing 100084, China; 2 Department of Physical Education, China Agricultural University, Beijing 100083, China; 3 China Institute of Sports and Health Science, Beijing Sport University, Beijing 100084, China; 4 Beijing Key Laboratory of Sports Performance and Skill Assessment, Beijing Sport University, Beijing 100084, China; 5 Key Laboratory for Performance Training & Recovery of General Administration of Sport, Beijing Sport University, Beijing 100084, China; 6 Department of Heavy Athletics Teaching and Research Section, School of Competitive Sports, Beijing Sport University, Beijing 100084, China; 7 China Shooting College, Hebei Provincial Sports Bureau, Shijiazhuang 050041, China

**Keywords:** metabolically unhealthy obesity, metabolic phenotype transition, exercise training, insulin resistance, amino acid metabolism

## Abstract

Background: Exercise training is known to improve metabolic health in individuals with obesity; however, its role in facilitating transitions between metabolically unhealthy obesity (MUO) and metabolically healthy obesity (MHO), as well as the associated metabolic adaptations, remains incompletely understood. Methods: A total of 84 young adults with obesity were enrolled and classified MUO (*n* = 55) or MHO (*n* = 29) based on baseline metabolic profiles. All participants completed an 8-week supervised exercise intervention. Anthropometric parameters, body composition, cardiometabolic markers, and VO_2_max were assessed before and after the intervention. Targeted metabolomics of 30 amino acid-related metabolites was performed in the MHO-R (remained MHO) and MUO-C (conversion from MUO to MHO) groups to explore exercise-associated metabolic adaptations following intervention. Results: Exercise training improved cardiometabolic risk profiles, including reductions in adiposity and improvements in insulin resistance-related markers. A proportion of participants transitioned from metabolically unhealthy to metabolically healthy obesity following the intervention. No significant between-group differences in amino acid metabolite changes were observed between MHO-R and MUO-C groups. Exploratory metabolomic analyses identified exercise-responsive alterations in amino acid-related metabolites, particularly involving arginine biosynthesis and histidine metabolism. Conclusions: Combined aerobic and resistance training is associated with improvements in metabolic health and phenotype transition in young adults with obesity. Observed alterations in arginine and histidine metabolism may reflect metabolic adaptations to exercise rather than transition-specific or causal mechanisms underlying phenotype conversion.

## 1. Introduction

Obesity has become a major global public health challenge. With the increasing prevalence of obesity, growing attention has been directed toward the heterogeneity of metabolic health within obese populations. Evidence indicates that individuals with similar body mass index (BMI) can exhibit markedly different metabolic profiles and risks of comorbidities [1]. Accordingly, obesity has been classified into two phenotypes based on cardiometabolic risk: MHO and MUO [2]. However, currently, no universally accepted criteria exist for defining MHO and MUO phenotypes. Previous studies have adopted varying combinations of metabolic syndrome-related indicators and cutoff values, contributing to heterogeneity across the literature [3,4,5]. Therefore, the present study adopted commonly used cardiometabolic risk factor criteria based on previous studies in Chinese populations [6].

Individuals with MUO have a substantially higher risk of cardiometabolic complications compared with those with MHO [7]. The MUO phenotype is characterized by impaired glucose and lipid metabolism and elevated blood pressure. Insulin resistance (IR) and non-alcoholic fatty liver disease (NAFLD) are considered key features underlying these differences [8], and their coexistence further increases the risk of type 2 diabetes mellitus (T2DM) and cardiovascular disease [9,10,11].

Importantly, the MUO phenotype is not irreversible, particularly in younger individuals who exhibit greater metabolic plasticity. Exercise interventions have been shown to improve glucose homeostasis and metabolic flexibility and may contribute to the transition from MUO to MHO [12,13]. However, the metabolic changes associated with this phenotype transition remain incompletely understood.

Recent research has extended beyond traditional lipid and glucose metabolism to highlight the role of amino acid metabolism in obesity-related metabolic dysfunction [14]. Elevated levels of branched-chain amino acids (BCAAs) and aromatic amino acids (AAAs) have been consistently associated with insulin resistance and increased risk of T2DM [15,16,17,18,19,20]. In contrast, reduced circulating levels of amino acids such as histidine, arginine, and glycine have also been linked to adverse metabolic profiles [21,22]. Despite these observations, the relationship between amino acid metabolism and metabolic phenotype transition in obesity remains unclear.

Exercise training, including aerobic exercise (AE) and resistance training (RT), has been shown to improve metabolic health through multiple pathways, including enhanced mitochondrial function and improved muscle metabolic capacity [23,24]. Combined exercise interventions may provide additional benefits compared with single-modality training. Therefore, the present study aimed to investigate whether exercise-induced improvements in metabolic health are accompanied by changes in amino acid metabolism in young adults with obesity undergoing metabolic phenotype transition. We hypothesized that individuals transitioning from MUO to MHO after exercise would exhibit distinct amino acid metabolic responses compared with those maintaining the MHO phenotype. Targeted metabolomics was used to explore amino acid metabolic adaptations associated with exercise-induced metabolic phenotype changes.

## 2. Methods

### 2.1. Participants

A total of 96 young adults with obesity (BMI ≥ 28 kg/m^2^; age 18–25 years) were recruited. Inclusion criteria were: (1) absence of known renal, hepatic, or gastrointestinal diseases that could affect metabolic function; (2) not pregnant or breastfeeding; and (3) no regular exercise habits or physical limitations affecting training participation. Baseline health status was evaluated using the Physical Activity Readiness Questionnaire (PAR-Q), electrocardiogram (ECG), and the Pittsburgh Sleep Quality Index (PSQI).

Metabolic health status was classified as MUO or MHO based on cardiometabolic criteria, including: systolic blood pressure (SBP) ≥ 130 mmHg or diastolic blood pressure (DBP) ≥ 85 mmHg; fasting blood glucose (FBG) ≥ 6.1 mmol/L or diagnosed type 2 diabetes mellitus under treatment; triglycerides (TG) ≥ 1.7 mmol/L; and high-density lipoprotein cholesterol (HDL-C) ≤ 1.04 mmol/L. Individuals presenting at least one cardiometabolic abnormality were classified as MUO, whereas those without any of these conditions were classified as MHO.

### 2.2. Exercise Intervention

All participants completed a supervised 8-week combined aerobic and resistance training program. Training sessions were performed three times per week on non-consecutive days, with each session lasting approximately 90 min and consisting of a 10-min warm-up, 40 min of resistance training, 30 min of aerobic exercise, and a 10-min cool-down.

Resistance training consisted of body weight exercises targeting major muscle groups, including the upper limbs, lower limbs, and core musculature. Exercises were performed in 4 sets of 12 repetitions with 2-min rest intervals between sets. The program was progressive, comprising two phases: weeks 1–4 (Phase 1) and weeks 5–8 (Phase 2), during which exercise difficulty and intensity were gradually increased.

Aerobic exercise consisted of track running performed at 50–75% of maximum heart rate (HRmax). Exercise intensity was progressively increased from 50–65% HRmax during weeks 1–4 to 60–75% HRmax during weeks 5–8. Heart rate was continuously monitored using a Polar heart rate monitoring system to ensure adherence to the prescribed intensity.

Attendance was recorded for each session, and adherence was defined as completion of at least 80% of the prescribed sessions. A detailed overview of the exercise protocol is provided in Supplementary Table S1.

### 2.3. Dietary Control

Participants were instructed to maintain their habitual dietary patterns and daily physical activity levels throughout the study period. Dietary intake was assessed every four weeks using a three-day food diary, including one training day, one low-activity day, and one rest day. Participants recorded all meals, snacks, and beverages using the Mint Health mobile application. Records with substantial deviations from baseline dietary patterns were excluded from the final analysis.

### 2.4. Sample Collection and Biochemical Analysis

Following a 12-h overnight fast, venous blood samples were collected by trained nurses and processed within 30 min. Samples were centrifuged at 3000 rpm for 20 min to separate plasma and serum, which were aliquoted and stored at −80 °C until analysis.

Biochemical parameters, including fasting blood glucose (FBG), triglycerides (TG), total cholesterol (TC), low-density lipoprotein cholesterol (LDL-C), and high-density lipoprotein cholesterol (HDL-C), were measured using an automated biochemical analyzer (UniCel DxC 800, Beckman Coulter, Brea, CA, USA). Fasting insulin (FINS) levels were determined using chemiluminescence immunoassay (UniCel DxI 800, Beckman Coulter, Brea, CA, USA). All measurements were performed according to standard laboratory protocols.

### 2.5. Targeted Metabolomics

Serum metabolites were analyzed using liquid chromatography–tandem mass spectrometry (LC–MS/MS) with an AB Sciex QTRAP 6500+ mass spectrometer (SCIEX, Framingham, MA, USA) coupled to a Shimadzu Nexera X2 UHPLC system (Shimadzu, Kyoto, Japan). Chromatographic separation was performed on a Waters UPLC BEH Amide column (1.7 μm, 2.1 × 100 mm; Milford, MA, USA). Metabolites were quantified using multiple reaction monitoring (MRM) mode based on extracted ion chromatogram (XIC) peak areas. Raw chromatographic data were processed by peak detection, peak integration, and manual inspection of extracted ion chromatograms to ensure correct metabolite identification and quantification. Metabolite peak areas were exported for subsequent preprocessing and statistical analysis.

Metabolite peak areas were normalized based on pooled quality control (QC) samples to reduce analytical variation across the analytical sequence. QC samples were used to monitor instrument stability and analytical reproducibility. All quantified metabolites were retained for multivariate analyses and visualization, whereas metabolites meeting the predefined statistical thresholds were used for downstream differential metabolite and pathway enrichment analyses.

Targeted metabolomics analysis was conducted in the MHO-R and MUO-C subgroups to explore metabolic adaptations associated with maintained or improved metabolic health following exercise intervention, rather than to provide a comprehensive assessment across all phenotype categories.

### 2.6. Additional Physiological Assessments

Blood pressure: Measured using an automated monitor CH-550 automated blood pressure monitor (Citizen Systems, Jiangmen, China) [25] after 5 min of seated rest. Two readings were taken 60 s apart from the left arm, and the average was recorded.

Body composition: Assessed via dual-energy X-ray absorptiometry (DEA; Lunar iDXA, GE HealthCare, Madison, WI, USA), with the subject lying supine during a 10–15-min scan. Regional fat distribution, including android fat mass (AFM) and gynoid fat mass (GFM), was also evaluated. The android region primarily represents abdominal adiposity, whereas the gynoid region encompasses the hips and thighs [26].

Maximal oxygen uptake (VO_2_max): Determined via an incremental exercise test on a Ergomedic 839E cycle ergometer (Monark Exercise AB, Monark, Sweden), starting at 50 W and increasing by 50 W every 3 min until volitional fatigue. Gas exchange was continuously monitored using a MetaLyzer 3B-R2 metabolic analyzer (Cortex, Biophysik GmbH, Leipzig, Germany).

**Figure 1:**
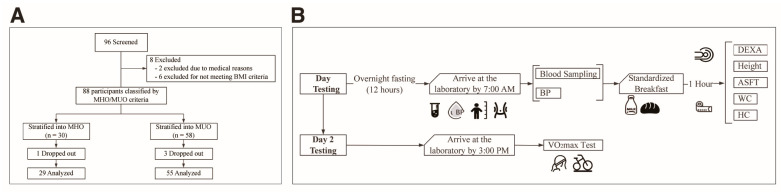
Study design and experimental procedures. (**A**) CONSORT Flow Diagram of Participant Enrollment, Allocation, and Analysis. A total of 96 participants were screened, among whom 88 were classified into either the MHO or MUO group according to metabolic health criteria. After accounting for dropouts, 29 MHO and 55 MUO individuals were included in the final analysis. (**B**) Schematic overview of the testing procedures. This diagram illustrates the sequence and components of physical and metabolic assessments conducted before and after the 8-week exercise intervention. On Day 1, participants arrived at the laboratory after a 12-h overnight fast. Blood sampling, blood pressure measurement, and anthropometric assessments were conducted following a standardized breakfast. On Day 2, participants returned to the laboratory in the afternoon for a VO_2_max test. Abbreviations: ASFT, abdominal subcutaneous fat thickness; WC, waist circumference; HC, hip circumference; BP, blood pressure.

The sequence of physiological and metabolic assessments conducted at baseline and post-intervention is illustrated in Figure 1B.

### 2.7. Statistical Analysis

Statistical analyses were performed using IBM SPSS Statistics (version 27) and R software (version 4.3.0). Continuous variables are presented as mean ± standard error (SE), unless otherwise specified. Group comparisons of phenotypic variables were conducted using generalized estimating equations (GEE), with statistical significance set at *p* < 0.05. Sex was included as a covariate in the analyses.

Metabolomic data were analyzed in R. Before statistical analysis, normalized metabolite data were log_2_-transformed to reduce skewness and improve the comparability of metabolite distributions. No missing values were observed in the quantified metabolite dataset. Principal component analysis (PCA) was used to visualize global metabolic patterns, followed by hierarchical clustering. For PCA and hierarchical clustering, log_2_-transformed data were further scaled before visualization. Within-group differences (post vs. pre) were assessed using the Wilcoxon signed-rank test, and between-group comparisons were conducted using the Mann–Whitney U test based on delta values. False discovery rate (FDR) correction was performed using the Benjamini–Hochberg method to account for multiple comparisons. Metabolites with FDR < 0.05 were considered significantly altered metabolites. Because no significant between-group differences in amino acid metabolite changes were observed after FDR correction, an additional exploratory combined analysis was performed to identify exercise-responsive metabolite alterations across the MHO-R and MUO-C groups.

Pathway enrichment analysis was performed using MetaboAnalyst based on the Kyoto Encyclopedia of Genes and Genomes (KEGG) human metabolic pathway database. Differential metabolites identified with FDR < 0.05 were used as input metabolites, while all quantified amino acid-related metabolites included in the targeted metabolomics panel were used as the background metabolite set. Pathway enrichment was evaluated using over-representation analysis combined with pathway topology analysis. Enriched pathways were considered significant based on FDR < 0.05, pathway impact > 0.1, and at least two matched metabolites.

Spearman correlation analysis was used to evaluate associations between differential metabolites and phenotypic variables.

### 2.8. Ethics Approval

The study was approved by the Ethics Committee of Beijing Sport University (approval number: 2024263H, approved on 16 July 2024) and conducted in accordance with the Declaration of Helsinki. Written informed consent was obtained from all participants prior to participation. The trial was registered with the Chinese Clinical Trial Registry (ChiCTR2400094932) on 30 December 2024.

## 3. Results

### 3.1. Baseline Characteristics

A total of 84 participants met the inclusion criteria and completed the 8-week exercise intervention and assessments (Figure 1A). Based on the predefined criteria, 29 participants (9 females) were classified as MHO and 55 (6 females) as MUO.

At baseline, no between-group differences were observed in anthropometric parameters (all *p* ≥ 0.05; Table 1). However, the proportion of females differed significantly between groups (*p* = 0.035). In contrast, several metabolic indicators differed significantly between groups. Specifically, SBP, DBP, FBG, and TG were higher in the MUO group than in the MHO group (all *p* < 0.05), whereas HDL-C did not differ between groups.

Additionally, HOMA-IR, insulin, and the HDL-C/TC ratio showed significant between-group differences (all *p* < 0.05). No differences were observed for TC, LDL-C, or VO_2_max.

### 3.2. Exercise Effects on Phenotype

Following the 8-week combined exercise intervention, both MHO and MUO groups showed significant improvements in anthropometric and body composition indicators (Tables S2 and S3). Specifically, body weight, BMI, percent body fat (PBF), AFM, GFM, ASFT, and AGR decreased significantly in both groups (all *p* < 0.05).

WC and HC decreased significantly in the MHO group (*p* < 0.05) but did not change in the MUO group. FFM increased slightly in the MHO group (*p* > 0.05) but decreased significantly in the MUO group (*p* < 0.05). WHR showed no significant change in either group. Significant time × group interactions were observed for body weight, FFM, and WHR (all *p* < 0.05).

For MHO/MUO classification indicators, SBP, DBP, and FBG decreased significantly in the MHO group (all *p* < 0.05), whereas TG increased (*p* < 0.05). In the MUO group, SBP and FBG decreased significantly (all *p* < 0.05), while DBP and TG did not change significantly. HDL-C remained unchanged in both groups. A significant time × group interaction was observed for TG (*p* < 0.05).

For other metabolic indicators, HOMA-IR, insulin, and TC decreased significantly in both groups (all *p* < 0.01), accompanied by an increase in the HDL-C/TC ratio (all *p* < 0.001). LDL-C decreased significantly only in the MUO group (*p* < 0.05). VO_2_max increased significantly in the MHO group (*p* < 0.01) but did not change in the MUO group. Significant time × group interactions were also observed for HOMA-IR and insulin (both *p* < 0.05).

### 3.3. Phenotype Transition Following Exercise Intervention

Following the 8-week exercise intervention, participants were reclassified according to metabolic health status. In the MHO group, 22 individuals remained metabolically healthy (MHO-R), whereas 7 transitioned to the MUO phenotype (MHO-C). In the MUO group, 29 participants remained metabolically unhealthy (MUO-R), while 26 transitioned to the MHO phenotype (MUO-C) (Table S4).

Anthropometric and body composition indicators showed significant Time × Group interaction effects for all variables except WHR (all *p* < 0.05; Table 2), indicating differential responses between MHO-R and MUO-C groups.

**Figure 2:**
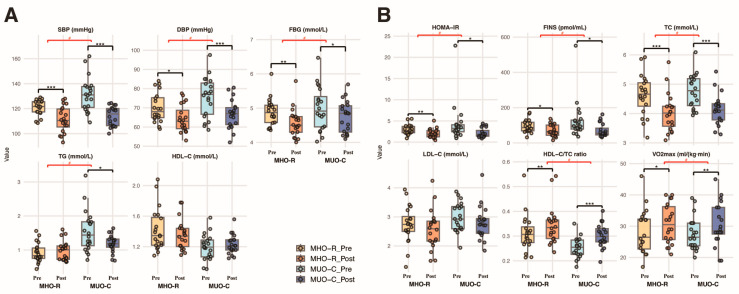
Pre- and post-intervention comparisons of metabolic health indicators in the MHO-R and MUO-C groups. (**A**) Classification indicators used to define MHO and MUO. (**B**) Additional metabolic health-related indicators. * indicates a significant within-group change (* *p* < 0.05, ** *p* < 0.01, *** *p* < 0.001); # indicates a significant time × group interaction (*p* < 0.05).

For MHO/MUO classification indicators, baseline differences were observed between MHO-R and MUO-C groups for SBP, DBP, TG, and HDL-C (all *p* < 0.05), whereas FBG did not differ between groups. Following the intervention, SBP, DBP, and FBG decreased significantly in both groups (all *p* < 0.05). TG decreased significantly only in the MUO-C group (*p* < 0.01), while HDL-C remained unchanged in both groups. Significant time × group interactions were observed for SBP, DBP, FBG, and TG (all *p* < 0.05; Table 2 and Figure 2).

For other metabolic indicators, HOMA-IR, insulin, and TC decreased significantly in both groups (all *p* < 0.05), whereas HDL-C/TC ratio and VO_2_max increased significantly (all *p* < 0.05). LDL-C showed no significant change. Time × group interactions were significant for all indicators except LDL-C (all *p* < 0.05; Table 2 and Figure 2).

### 3.4. Metabolomic Responses

**Figure 3:**
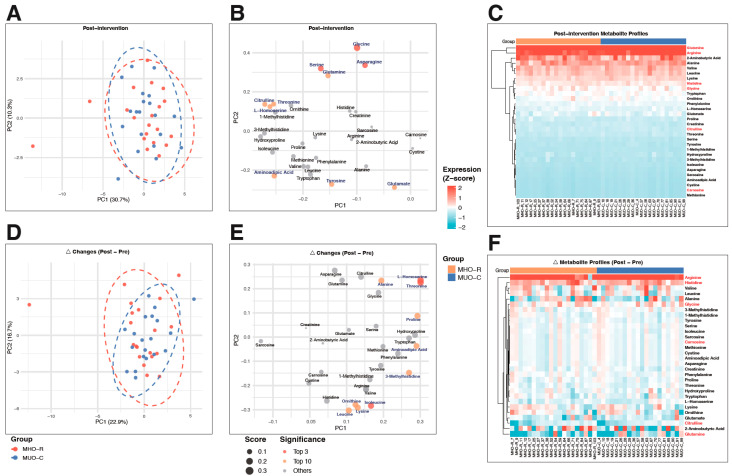
Post-intervention amino acid profiles and changes (Δ) in the MHO-R and MUO-C groups. (**A**,**D**) PCA score plots: Each point represents an individual, with red and blue indicating the MHO-R and MUO-C groups, respectively. Ellipses represent 95% confidence intervals. (**B**,**E**) PCA loading plots: Dot size reflects each metabolite’s squared loading score (i.e., contribution to the principal components). Colors denote relative importance: top 3 (red), ranks 4–10 (orange), others (gray). (**C**,**F**) Z-score heatmaps of post-intervention metabolite levels and changes (Δ). Color scale reflects relative abundance (red: higher; white: average; blue: lower). Metabolites labeled in red show significant within-group changes (FDR < 0.05).

PCA of 30 amino acid metabolites after the intervention showed that PC1 and PC2 explained 30.7% and 10.3% of the total variance, respectively (Figure 3A). The PCA score plot demonstrated substantial overlap between the MHO-R and MUO-C groups, with no clear separation. PCA based on metabolite changes (Post–Pre) similarly showed no separation between groups, with PC1 and PC2 explaining 22.9% and 16.7% of the variance, respectively (Figure 3D). Hierarchical clustering heatmaps of post-intervention metabolite levels and metabolite changes also showed similar clustering patterns between groups (Figure 3C,F). Consistently, between-group comparisons of metabolite changes revealed no significant differences (all FDR > 0.05; Table S5).

**Figure 4:**
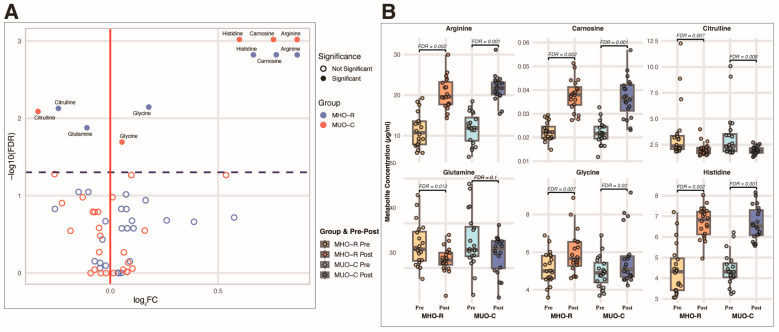
Metabolite changes in the MHO-R and MUO-C groups pre- and post- intervention. (**A**) Combined volcano plot showing differential metabolite responses (Post vs. Pre) within the MHO-R and MUO-C groups. Each point represents a metabolite; those with FDR < 0.05 are displayed as filled markers, with annotated names for selected significant metabolites. (**B**) Box plots of representative differential metabolites with significant within-group changes (FDR < 0.05). Data are visualized as box plots showing the median and interquartile range (IQR).

Therefore, subsequent analyses focused primarily on exercise-responsive metabolite changes rather than phenotype-specific between-group differences. Within-group analyses identified six significantly altered metabolites in the MHO-R group (FDR < 0.05), including increased arginine, carnosine, histidine, and glycine, and decreased citrulline and glutamine. Similarly, five metabolites were significantly altered in the MUO-C group, including increased arginine, carnosine, histidine, and glycine, and decreased citrulline (Table 3 and Figure 4). Complete metabolite-level statistics are provided in Table S6.

Given the absence of significant between-group differences in metabolite changes, an exploratory combined analysis was performed by pooling the MHO-R and MUO-C groups. This analysis identified 12 significantly altered metabolites (FDR < 0.05; Table S8), suggesting a shared exercise-responsive amino acid metabolic profile across the two groups.

### 3.5. Mechanistic Pathway

**Figure 5:**
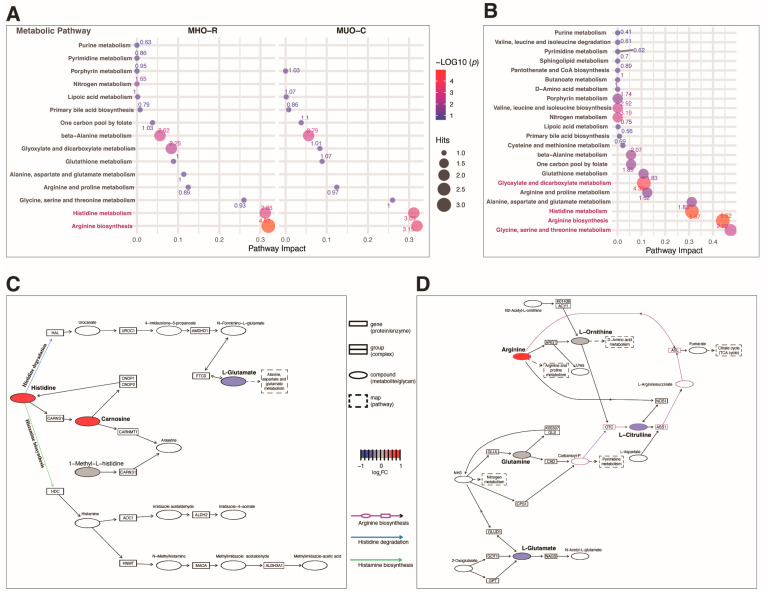
Pathway enrichment analysis and representative metabolic pathways altered after exercise intervention**.** (**A**) Pathway enrichment bubble plot based on differential amino acid metabolites in the MHO-R and MUO-C groups. (**B**) Pathway enrichment using combined data (MHO-R + MUO-C). Each bubble represents a KEGG pathway; bubble size indicates the number of matched metabolites (Hits), and color intensity corresponds to statistical significance (–log_10_ *p*-value). Significantly enriched pathways (FDR < 0.05) are labeled in purple. (**C**) Arginine biosynthesis pathway and (**D**) histidine metabolism pathway visualized using the pathview R package. Node color represents the direction and magnitude of change in log_2_-transformed fold change (log_2_FC): red indicates upregulation (log_2_FC = +1), blue indicates downregulation (log_2_FC = –1), and gray indicates no change (log_2_FC = 0). Only metabolites with FDR < 0.05 are highlighted.

KEGG pathway enrichment analysis was performed based on the differential metabolites identified in each group. In the MHO-R group, arginine biosynthesis was significantly enriched (Hits = 3, Impact = 0.319, FDR < 0.001). In the MUO-C group, arginine biosynthesis (Hits = 2, Impact = 0.319, FDR = 0.037) and histidine metabolism (Hits = 2, Impact = 0.311, FDR = 0.037) were significantly enriched (Table 4, Figure 5A). Complete pathway enrichment results are provided in Table S7. Based on the exploratory combined dataset, four enriched KEGG pathways were identified (Table S9, Figure 5B), including arginine biosynthesis, histidine metabolism, glyoxylate and dicarboxylate metabolism, and glycine, serine and threonine metabolism. Mechanistic pathways for histidine metabolism and arginine biosynthesis are presented in Figure 5C,D.

### 3.6. Correlation Analysis

Spearman correlation analysis was performed to examine the associations between changes (ΔPost–Pre) in 12 significantly altered metabolites and corresponding changes in phenotypic indicators. Changes in arginine were negatively correlated with changes in FBG, TC, and LDL-C, and positively correlated with changes in the HDL-C/TC ratio (all *p* < 0.05). In contrast, changes in histidine were not associated with any phenotypic indicators. Correlations for the remaining metabolites are presented in Figure S1.

## 4. Discussion

This study evaluated the effects of an 8-week combined aerobic and resistance training program on metabolic phenotypes in young adults with obesity and investigated potential mechanisms using targeted amino acid metabolomics. The main findings were that exercise improved anthropometric and metabolic parameters and promoted the transition from the MUO to the MHO phenotype. Interestingly, although no significant improvement in VO_2_max was observed in the overall MUO group following intervention, significant improvements in cardiorespiratory fitness were observed in the MUO-C subgroup. This finding may suggest that improvements in metabolic phenotype and cardiorespiratory fitness may not occur uniformly among individuals with metabolically unhealthy obesity [27].

Contrary to our initial hypothesis, the amino acid metabolomic responses following exercise intervention appeared broadly similar between the MHO-R and MUO-C groups. While exercise-induced improvements in phenotypic outcomes differed between groups, amino acid metabolic alterations showed a more consistent adaptive pattern. This may suggest that exercise-responsive amino acid remodeling reflects generalized metabolic adaptations accompanying exercise intervention rather than phenotype-transition-specific signatures. Interestingly, although both arginine- and histidine-related pathways were enriched following exercise intervention, only arginine-related metabolite changes were significantly correlated with glycemic and lipid indicators. This difference may reflect the more direct involvement of arginine metabolism in nitric oxide production, vascular function, and glucose–lipid metabolic regulation [28], whereas histidine-related alterations may represent broader exercise-associated metabolic adaptations that are less directly linked to phenotypic metabolic markers.

Metabolomic analyses revealed consistent alterations in amino acid profiles following the intervention, with enrichment of pathways related to arginine biosynthesis and histidine metabolism. Furthermore, changes in arginine were associated with improvements in lipid and glycemic indicators, suggesting that these amino acid metabolic alterations may reflect exercise-responsive adaptations related to metabolic improvement rather than phenotype-specific mechanisms underlying phenotype transition.

### 4.1. The Role of Arginine Metabolism

L-arginine (L-Arg), a substrate for nitric oxide synthase (NOS), is involved in nitric oxide (NO) production and plays a key role in vascular function, inflammation regulation, and insulin signaling. Previous studies have shown that NO enhances glucose uptake in skeletal muscle and reduces inflammation, partly through activation of the IRS–PI3K–Akt signaling pathway [29,30,31,32]. In addition, exercise has been reported to increase endothelial NOS (eNOS) activity and NO production, thereby improving tissue perfusion and insulin sensitivity [33,34,35,36]. Mechanistically, NO can promote downstream activation of insulin signaling via PKB/Akt, contributing to enhanced glucose uptake and reduced pro-inflammatory responses [28]. In the present study, L-Arg levels increased following the intervention, and changes in L-Arg were associated with improvements in metabolic indicators, suggesting that activation of the L-Arg–NO pathway may be involved in exercise-induced metabolic adaptations.

In addition to NO production, L-Arg metabolism generates downstream metabolites such as ornithine, citrulline, and polyamines, which are involved in cell proliferation, oxidative stress regulation, and lipid metabolism (Figure 5D) [37,38,39,40]. Previous studies have shown that L-Arg supplementation can reduce blood pressure and platelet aggregation, enhance pancreatic β-cell function, and improve glucose and lipid metabolism in individuals with metabolic disorders [28,29,30,31]. Consistent with these findings, increases in L-Arg in the present study were associated with improvements in FBG, TC, LDL-C, and the HDL-C/TC ratio, further supporting a potential role of L-Arg metabolism in exercise-induced metabolic improvements.

Previous studies have reported that long-term L-Arg supplementation improves glucose tolerance and may delay the progression to type 2 diabetes, potentially through mechanisms involving reduced oxidative stress and improved endothelial function [38,41,42,43]. Randomized trials have also shown that L-Arg, in combination with dietary control and exercise, can reduce fat mass and improve insulin sensitivity, possibly through enhanced eNOS activity and NO bioavailability [44]. Systematic reviews further support the role of L-Arg in improving fasting glucose and insulin levels in individuals with impaired glucose metabolism [45]. However, potential adverse effects should be considered, as excessive L-Arg may contribute to the formation of advanced glycation end-products (AGEs), which are associated with vascular inflammation and damage [46].

### 4.2. Histidine Metabolism and Downstream Signaling

L-histidine (L-His) represents another exercise-responsive metabolic pathway and can be metabolized into carnosine and histamine (Figure 5C). Through condensation with β-alanine, L-His forms carnosine and anserine, which contribute to buffering capacity and antioxidative defense in skeletal muscle [47,48,49]. In the present study, L-His and carnosine levels increased following the intervention, and pathway enrichment analysis further supported the involvement of histidine metabolism in exercise-induced metabolic adaptation.

L-His can also be decarboxylated by histidine decarboxylase (HDC) to produce histamine, which exerts physiological effects through H_1_ and H_2_ receptors and has been implicated in vasodilation, energy metabolism, and central regulation [50,51,52]. Exercise has been reported to increase histamine release in peripheral tissues and the central nervous system, potentially influencing muscle perfusion, inflammatory responses, and appetite regulation [51,53,54,55]. In addition, histidine and its derivatives have been associated with inflammation, insulin sensitivity, and energy balance in individuals with metabolic disorders [56,57,58].

Previous studies have shown that L-His supplementation can reduce FBG and circulating inflammatory markers, possibly through modulation of inflammatory signaling pathways [57]. Histamine has also been linked to central regulation of energy intake via hypothalamic pathways [59,60,61]. However, in the present study, changes in L-His were not associated with improvements in metabolic indicators. Together with the enrichment of histidine metabolism pathways, these findings suggest that histidine-related metabolism may contribute to exercise-induced metabolic regulation, although its role appears to be less direct compared with that of L-Arg [51].

### 4.3. Exercise-Responsive Amino Acid Metabolic Adaptations

Collectively, the present findings suggest that combined exercise intervention was associated with improvements in metabolic phenotype accompanied by coordinated alterations in amino acid metabolism. In particular, pathways related to L-Arg metabolism were significantly enriched following exercise intervention. Given the established roles of L-Arg metabolism in nitric oxide production, vascular function, and insulin signaling, these alterations may reflect exercise-related metabolic remodeling associated with improved metabolic regulation [28,29,30,31]. In the present study, changes in arginine-related metabolites were also associated with glycemic and lipid-related indicators, further supporting the potential involvement of endogenous L-Arg metabolism in exercise-responsive metabolic adaptation.

Histidine metabolism was also identified as a significantly enriched pathway following exercise intervention. Previous studies have shown that L-His supplementation may reduce fasting blood glucose and circulating inflammatory markers, potentially through modulation of inflammatory signaling pathways. In addition, histamine has been implicated in the central regulation of energy intake through hypothalamic pathways [50,51,52]. However, unlike L-Arg-related metabolites, changes in L-His were not significantly associated with phenotypic metabolic indicators in the present study. These findings suggest that histidine-related metabolic alterations may represent broader exercise-associated metabolic adaptations rather than direct regulators of phenotypic metabolic improvement.

Importantly, although MHO-R and MUO-C groups exhibited different phenotypic responses following intervention, amino acid metabolic responses were largely comparable between groups, and no significant between-group metabolite differences were observed after FDR correction. Therefore, the enriched arginine biosynthesis and histidine metabolism pathways should be interpreted as shared exercise-responsive metabolic adaptations rather than phenotype-specific mechanisms directly underlying MUO-to-MHO transition [62]. Compared with previous studies primarily focusing on exogenous supplementation of L-Arg and L-His, the present findings provide exploratory evidence that endogenous amino acid metabolic remodeling may contribute to exercise-associated metabolic improvement and may represent potential targets for future mechanistic studies.

It should also be noted that, although dietary intake was monitored throughout the intervention and participants were instructed to maintain their habitual dietary patterns, subtle dietary variations, particularly in protein intake, cannot be completely excluded and may have partially influenced circulating amino acid profiles.

### 4.4. Limitations and Future Directions

Several limitations should be acknowledged. First, the absence of a non-exercise control group limited our ability to distinguish exercise-induced changes from potential time-related or lifestyle-related effects. Second, the overall sample size and subgroup sample sizes were relatively small, which may have limited the statistical power to detect between-group phenotypic and metabolomic differences. Third, targeted metabolomics was used, restricting the coverage of broader metabolic pathways and limiting mechanistic interpretation. Fourth, metabolomic analyses were conducted in selected subgroups rather than across all phenotype categories, including MHO-C and MUO-R, and the categorical classification of metabolic health may oversimplify the continuum of metabolic risk.

Future studies with larger sample sizes and comprehensive metabolomic profiling are warranted. Mechanistic investigations using cell and animal models are also needed to elucidate the roles of key pathways, including arginine and histidine metabolism, in exercise-induced metabolic adaptations.

## 5. Conclusions

This study demonstrates that combined aerobic and resistance training improves metabolic health and is associated with a transition from metabolically unhealthy to metabolically healthy obesity in young adults. Exercise-induced changes in amino acid metabolism, particularly in arginine and histidine-related pathways, may reflect shared metabolic adaptations to exercise rather than causal or transition-specific mechanisms underlying phenotype conversion. These findings support the role of structured exercise interventions in improving metabolic health and provide a basis for future studies exploring exercise-responsive metabolic remodeling.

## Figures and Tables

**Table 1:** Baseline demographic, physical, and metabolic characteristics.

Variable	MHO (*n* = 29)	MUO (*n* = 55)	*p*-Value
Sex (F/M, %)	9/20(31.0/69.0)	6/49(10.9/89.1)	0.035 †
Age (years)	19.61 ± 1.43	19.59 ± 1.42	0.916
Weight (kg)	95.45 ± 1.54	96.47 ± 1.60	0.112
BMI (kg/m^2^)	32.02 ± 0.41	32.31 ± 0.43	0.156
PBF (%)	37.67 ± 0.54	37.51 ± 0.58	0.840
WC (cm)	101.45 ± 1.83	99.69 ± 1.44	0.279
HC (cm)	112.24 ± 1.34	111.70 ± 0.88	0.367
WHR	0.89 ± 0.01	0.89 ± 0.01	0.433
SBP (mmHg)	120.25 ± 1.07	132.44 ± 1.50	<0.001
DBP (mmHg)	67.96 ± 1.29	75.96 ± 1.16	<0.001
FBG (mmol/L)	4.81 ± 0.06	5.02 ± 0.08	0.010
TG (mmol/L)	0.92 ± 0.06	1.45 ± 0.11	<0.001
HDL-C (mmol/L)	1.24 ± 0.03	1.20 ± 0.03	0.147
HOMA-IR	2.81 ± 0.22	3.96 ± 0.49	0.001
Insulin (pmol/L)	89.12 ± 6.60	120.97 ± 12.09	0.001
TC (mmol/L)	4.38 ± 0.14	4.53 ± 0.09	0.731
LDL-C (mmol/L)	2.59 ± 0.13	2.72 ± 0.08	0.848
HDL-C/TC ratio	0.27 ± 0.01	0.26 ± 0.01	0.049
VO_2_max (ml/(kg·min))	25.60 ± 0.90	25.59 ± 0.57	0.455

Note: Values are presented as mean ± SE, unless otherwise specified. *p*-values indicate between-group comparisons (MHO vs. MUO) at baseline. † *p*-value for sex was calculated using Fisher’s Exact Test. Sex is reported as the number of females/males (F/M) with corresponding percentages.

**Table 2:** Changes (Δ) in physical and metabolic characteristics (MHO-R vs. MUO-C) after exercise intervention.

Variable	MHO-R (n = 22)	MUO-C (n = 26)	*p* (Time × Group)
	(F/M = 8/14)	(F/M = 3/23)	
Anthropometric and body composition indicators			
Weight (kg)	−2.05 ± 0.75 **	−3.71 ± 0.65 ***	<0.001
BMI (kg/m^2^)	−0.73 ± 0.25 **	−1.22 ± 0.21 ***	<0.001
PBF (%)	−2.03 ± 0.35 ***	−2.26 ± 0.39 ***	<0.001
FFM (kg)	0.47 ± 0.31	−0.22 ± 0.31	0.003
AFM (kg)	−0.31 ± 0.08 ***	−0.45 ± 0.08 ***	<0.001
GFM (kg)	−0.42 ± 0.09 ***	−0.52 ± 0.10 ***	<0.001
ASFT (mm)	−1.86 ± 1.00	−2.68 ± 0.79 ***	0.002
WC (cm)	−7.71 ± 3.45 *	−4.81 ± 2.97	0.019
HC (cm)	−5.64 ± 2.32 *	−3.42 ± 1.97	0.026
WHR	−0.02 ± 0.02	−0.01 ± 0.02	0.274
AGR	−0.01 ± 0.01	−0.03 ± 0.01 ***	0.002
Indicators for defining MHO and MUO			
SBP (mmHg)	−8.25 ± 1.96 ***	−16.35 ± 2.72 ***	<0.001
DBP (mmHg)	−3.50 ± 1.65 *	−9.00 ± 1.39 ***	<0.001
FBG (mmol/L)	−0.26 ± 0.08 **	−0.32 ± 0.14 *	<0.001
TG (mmol/L)	0.10 ± 0.06	−0.26 ± 0.11 *	0.002
HDL-C (mmol/L)	−0.03 ± 0.03	0.00 ± 0.03	0.179
Other metabolic health-related indicators			
HOMA-IR	−0.72 ± 0.27 **	−1.93 ± 0.80 *	0.005
FINS (pmol/L)	−18.87 ± 8.27 *	−48.58 ± 19.12 *	0.009
TC (mmol/L)	−0.45 ± 0.11 ***	−0.59 ± 0.11 ***	<0.001
LDL-C (mmol/L)	−0.07 ± 0.10	−0.14 ± 0.09	0.296
HDL-C/TC	0.03 ± 0.01 **	0.04 ± 0.01 ***	<0.001
VO_2_max (mL/(kg·min))	1.88 ± 0.87 *	2.50 ± 0.84 **	0.003

Note: All values are expressed as mean difference ± SE, calculated as post-intervention minus pre-intervention values. * indicates a significant pre-to-post change within the MHO-R or MUO-C group (* *p* < 0.05, ** *p* < 0.01, *** *p* < 0.001). *p* (Time × Group) refers to the *p*-value of the interaction term. AGR, arginine-to-glycine ratio.

**Table 3:** Differential amino acid metabolites in MHO-R and MUO-C groups (Pre vs. Post).

Group	Metabolite	FC	log_2_FC	*p*-Value	FDR	Direction
MHO-R	Arginine	1.824	0.867	<0.001	0.002	↑
MHO-R	Carnosine	1.705	0.770	<0.001	0.002	↑
MHO-R	Histidine	1.584	0.664	<0.001	0.002	↑
MHO-R	Glycine	1.132	0.178	0.001	0.007	↑
MHO-R	Citrulline	0.846	−0.241	0.001	0.007	↓
MHO-R	Glutamine	0.928	−0.108	0.003	0.013	↓
MUO-C	Arginine	1.823	0.867	<0.001	0.001	↑
MUO-C	Carnosine	1.690	0.757	<0.001	0.001	↑
MUO-C	Histidine	1.514	0.599	<0.001	0.001	↑
MUO-C	Citrulline	0.793	−0.335	0.001	0.008	↓
MUO-C	Glycine	1.038	0.054	0.003	0.020	↑

Note: Differential amino acid metabolites were identified by comparing pre- and post-intervention data within each group (MHO-R and MUO-C). FC, fold change (Post/Pre); log_2_FC, log_2_-transformed fold change; *p*-value, raw *p*-value from Wilcoxon signed-rank test; FDR, false discovery rate adjusted *p*-value. Direction (↑/↓) indicates up- or downregulation. Metabolites with FDR < 0.05 were considered statistically significant.

**Table 4:** Significantly enriched amino acid metabolic pathways following exercise intervention in the MHO-R and MUO-C groups.

Group	Pathway	Hits	FDR	Impact
MHO-R	Arginine biosynthesis	3	9.6 × 10^−4^	0.319
MUO-C	Histidine metabolism	2	0.037	0.311
MUO-C	Arginine biosynthesis	2	0.037	0.319

Note: This table summarizes significantly enriched amino acid metabolic pathways identified from within-group metabolite changes (Post vs. Pre) following exercise intervention. Pathway enrichment analysis was performed using the KEGG database and MetaboAnalyst. Enriched pathways were defined based on the predefined criteria of FDR < 0.05, pathway impact > 0.1, and Hits ≥ 2. Hits indicates the number of matched metabolites identified within each pathway, and Impact indicates the pathway topology impact score.

## Data Availability

The datasets generated and/or analyzed during the current study are available from the corresponding author on reasonable request for academic purposes.

## Supplementary Materials

The following supporting information can be downloaded at: https://www.mdpi.com/article/10.3390/nu18121956/s1, Figure S1: Spearman correlation analysis between differential amino acid metabolites and phenotypic indicators; Table S1: Overview of the eight-week combined aerobic and resistance training program; Table S2: Physical and metabolic indicators pre- and post-intervention in the MHO and MUO groups; Table S3: Changes (Δ) in physical and metabolic indicators (MHO vs. MUO) after exercise intervention; Table S4: Physical and metabolic indicators pre- and post-exercise intervention in the MHO-R, MHO-C, MUO-R, and MUO-C groups; Table S5: Between-group comparison of changes (Δ) in amino acid metabolites between the MHO-R and MUO-C groups; Table S6: Within-group changes in amino acid metabolites after exercise intervention in the MHO-R and MUO-C groups (Pre vs. Post); Table S7: Pathway enrichment analysis of exercise-responsive metabolites in the MHO-R and MUO-C groups (Pre vs. Post); Table S8: Amino acid metabolite changes identified after combining the MHO-R and MUO-C groups (Pre vs. Post); Table S9: Pathway enrichment analysis after combining the MHO-R and MUO-C groups (Pre vs. Post).
