# Supplementary material for: Combined Aerobic and Resistance Training Improves Metabolic Health and Is Associated with Arginine and Histidine Metabolic Changes During the Transition from Metabolically Unhealthy to Metabolically Healthy Obesity in Young Adults
Source: Nutrients. 2026 Jun 17;18(12):1956. doi: 10.3390/nu18121956 (PMC13305316; doi:10.3390/nu18121956)
Supplement: Supplementary file 1 [file nutrients-18-01956-s001.zip › nutrients-4350639-supplementary.pdf]

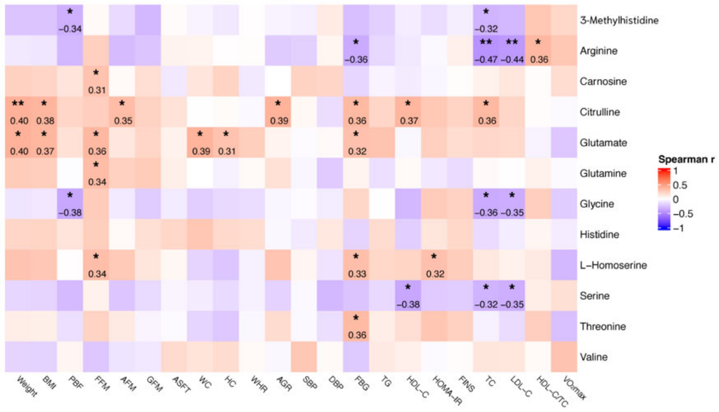


**Figure S1. Spearman correlation analysis between differential amino acid metabolites and phenotypic indicators.** *Heatmap showing the correlations between 12 significantly altered metabolites and phenotypic indicators (derived from combined data). Colors represent correlation direction and magnitude (red: positive, blue: negative). Spearman's correlation coefficients (r) are displayed below the heatmap. Significance levels are indicated by asterisks: *p < 0.05, **p < 0.01.*

**Table S1. Overview of the eight-week combined aerobic and resistance training program.**

| | | RT (40 min) | AE (30 min) |
|---|---|---|---|
| Phase | **Phase 1 (1-4 weeks)** | - Kneeling push-ups<br>- Standard squats<br>- Back extension<br>- Bridge with diagonal arm lift<br>- Supine crunches | 400m track running (50-65% HRmax) |
| | **Phase 2 (5-8 weeks)** | - Standard push-ups<br>- Squat jumps<br>- Back extension<br>- Bridge with diagonal arm lift<br>- Supine alternate knee tucks | 400m track running (60-75% HRmax) |
| **T (Type)** | | Bodyweight RT | Aerobic Running |
| **I (Intensity)** | | 4 sets × 12 reps (2-min inter-set rest) | 50-75% HRmax |
| **F (Frequency)** | 3 sessions/week (non-consecutive days) | | |
| **T (Time)** | 90 min/session (11:15 am–12:45 pm): warm-up (10 min) → RT (40 min) → AE (30 min) → cool-down (10 min) | | |

*Note: The 8-week combined training program was designed according to the F.I.T.T principle: Frequency (3 sessions/week), Intensity (50–75% HRmax for AE; 4 × 12 reps for RT), Time (90 min/session), and Type (bodyweight RT and aerobic running). The program was implemented in two progressive phases (Weeks 1–4 and 5–8).*

**Table S2. Physical and metabolic indicators pre- and post-intervention in the MHO and MUO groups.**

| Variable | MHO (n = 29) (F/M = 9/20) | | MUO (n = 55) (F/M = 6/49) | |
|---|---|---|---|---|
| | Pre | Post | Pre | Post |
| **Anthropometric and body composition indicators** | | | | |
| Weight (kg) | 98.45±1.54 | 96.13±1.66 | 99.61±1.6 | 95.31±1.64 |
| BMI | 32.82±0.41 | 32.00±0.46 | 33.15±0.43 | 31.75±0.45 |
| PBF (%) | 38.73±0.54 | 36.53±0.66 | 38.65±0.58 | 36.74±0.62 |
| FFM (kg) | 60.17±0.85 | 60.60±0.93 | 60.78±0.90 | 60.05±0.95 |
| AFM (kg) | 3.66±0.13 | 3.32±0.13 | 3.75±0.12 | 3.26±0.12 |
| GFM (kg) | 5.86±0.15 | 5.41±0.17 | 5.91±0.17 | 5.38±0.17 |
| ASFT (mm) | 32.73±0.82 | 30.96±0.71 | 33.94±0.56 | 32.07±0.87 |
| WC (cm) | 105.03±1.83 | 98.65±1.73 | 102.51±1.44 | 102.31±1.54 |
| HC (cm) | 114.86±1.34 | 110.39±1.12 | 113.42±0.88 | 111.52±1.15 |
| WHR | 0.91±0.01 | 0.89±0.01 | 0.90±0.01 | 0.92±0.01 |
| AGR | 0.63±0.02 | 0.61±0.01 | 0.64±0.01 | 0.61±0.01 |
| **Indicators for defining MHO and MUO** | | | | |
| SBP (mmHg) | 122.34±1.07 | 115.47±1.38 | 135.38±1.5 | 130.01±1.61 |
| DBP (mmHg) | 70.48±1.29 | 67.44±1.00 | 78.23±1.16 | 75.97±1.57 |
| FBG (mmol/L) | 4.93±0.06 | 4.72±0.07 | 5.18±0.08 | 4.89±0.09 |
| TG (mmol/L) | 1.03±0.06 | 1.16±0.05 | 1.67±0.11 | 1.46±0.09 |
| HDL-C (mmol/L) | 1.30±0.03 | 1.27±0.02 | 1.25±0.03 | 1.26±0.03 |
| **Other metabolic health-related indicators** | | | | |
| HOMA-IR | 3.24±0.22 | 2.51±0.19 | 4.91±0.49 | 2.93±0.47 |
| FINS (pmol/mL) | 102.05±6.60 | 82.62±5.57 | 144.66±12.09 | 89.95±10.95 |
| TC (mmol/L) | 4.66±0.14 | 4.14±0.09 | 4.71±0.09 | 4.13±0.11 |
| LDL-C (mmol/L) | 2.84±0.13 | 2.73±0.09 | 2.87±0.08 | 2.71±0.10 |
| HDL-C/TC | 0.29±0.01 | 0.32±0.01 | 0.27±0.01 | 0.31±0.01 |
| $VO_2max$ (ml/(kg·min) | 27.37±0.90 | 29.49±0.77 | 26.71±0.57 | 27.38±0.82 |

*Note: All values are expressed as mean ± standard error (SE).*

**Table S3. Changes (Δ) in physical and metabolic indicators (MHO vs MUO) after exercise intervention.**

| Variable | MHO (n = 29) (F/M = 9/20) | MUO (n = 55) (F/M = 6/49) | *p* (Time × Group) |
|---|---|---|---|
| **Anthropometric and body composition indicators** | | | |
| Weight (kg) | -2.32±0.65*** | -4.3±0.51*** | 0.033 |
| BMI | -0.82±0.21*** | -1.4±0.17*** | 0.056 |
| PBF (%) | -2.21±0.31*** | -1.92±0.24*** | 0.496 |
| FFM (kg) | 0.43±0.28 | -0.73±0.3* | 0.010 |
| AFM (kg) | -0.34±0.07*** | -0.49±0.05*** | 0.126 |
| GFM (kg) | -0.45±0.08*** | -0.53±0.07*** | 0.483 |
| ASFT (mm) | -1.76±0.85* | -1.87±0.83* | 0.932 |
| WC (cm) | -6.39±2.26** | -0.21±2.29 | 0.050 |
| HC (cm) | -4.47±1.74* | -1.9±1.57 | 0.271 |
| WHR | -0.02±0.01 | 0.01±0.01 | 0.038 |
| AGR | -0.02±0.01* | -0.03±0.01*** | 0.355 |
| **Indicators for defining MHO and MUO** | | | |
| SBP (mmHg) | -6.87±1.48*** | -5.37±1.7** | 0.502 |
| DBP (mmHg) | -3.04±1.39* | -2.26±1.52 | 0.723 |
| FBG (mmol/L) | -0.21±0.07** | -0.29±0.1** | 0.497 |
| TG (mmol/L) | 0.13±0.06* | -0.22±0.13 | 0.023 |
| HDL-C (mmol/L) | -0.03±0.02 | 0.01±0.02 | 0.308 |
| **Other metabolic health-related indicators** | | | |
| HOMA-IR | -0.73±0.24** | -1.98±0.49*** | 0.024 |
| FINS (pmol/mL) | -19.44±7.4** | -54.71±13.24*** | 0.025 |
| TC (mmol/L) | -0.52±0.12*** | -0.58±0.09*** | 0.655 |
| LDL-C (mmol/L) | -0.12±0.11 | -0.16±0.08* | 0.723 |
| HDL-C/TC | 0.03±0.01*** | 0.04±0.01*** | 0.339 |
| $VO_2max$ (ml/(kg·min) | 2.12±0.78** | 0.66±0.72 | 0.199 |

*Note: All values are expressed as mean ± standard error (SE), calculated as post-intervention minus pre-intervention values. * indicates a significant pre-to-post change within the MHO or MUO group (*p < 0.05, **p < 0.01, ***p < 0.001). p (Time × Group) denotes the interaction p-value. AGR, arginine-to-glycine ratio.*

**Table S4. Physical and metabolic indicators pre- and post-exercise intervention in the MHO-R, MHO-C, MUO-R, and MUO-C groups.**

| Variable | MHO-R (n = 22) (F/M = 8/14) | | MUO-C (n = 26) (F/M = 3/23) | | MHO-C (n = 7) (F/M = 1/6) | | MUO-R (n = 29) (F/M = 3/26) | |
|---|---|---|---|---|---|---|---|---|
| | **Pre** | **Post** | **Pre** | **Post** | **Pre** | **Post** | **Pre** | **Post** |
| **Anthropometric and body composition indicators** | | | | | | | | |
| Weight (kg) | 93.53±3.33 | 91.48±3.67 | 99.51±2.50 | 95.80±2.56 | 92.5±3.74 | 88.5±3.9 | 104.88±2.41 | 100.79±2.46 |
| BMI | 32.39±0.90 | 31.66±1.02 | 32.76±0.75 | 31.54±0.77 | 32.17±0.77 | 30.78±0.91 | 33.98±0.65 | 32.65±0.68 |
| PBF (%) | 39.29±1.33 | 37.25±1.55 | 37.75±0.96 | 35.49±1.07 | 40.15±1.60 | 37.69±2.07 | 38.7±0.70 | 36.89±0.75 |
| FFM (kg) | 56.38±1.84 | 56.84±2.03 | 61.65±1.35 | 61.44±1.40 | 55.29±2.30 | 55.06±2.59 | 64.06±1.29 | 63.35±1.35 |
| AFM (kg) | 3.53±0.29 | 3.23±0.30 | 3.60±0.18 | 3.15±0.19 | 3.51±0.35 | 3.00±0.31 | 4.02±0.18 | 3.55±0.17 |
| GFM (kg) | 5.78±0.37 | 5.37±0.41 | 5.78±0.29 | 5.26±0.27 | 5.55±0.32 | 4.94±0.36 | 6.16±0.23 | 5.66±0.23 |
| ASFT (mm) | 33.24±1.12 | 31.39±1.16 | 32.97±0.88 | 30.29±1.06 | 32.83±1.84 | 33.64±2.87 | 34.38±0.72 | 31.97±0.91 |
| WC (cm) | 106.27±2.23 | 98.57±3.19 | 103.44±2.22 | 98.63±1.71 | 101.21±2.19 | 100.43±2.87 | 101.66±1.85 | 102.83±1.79 |
| HC (cm) | 115.48±1.44 | 109.84±1.8 | 114.15±1.37 | 110.73±1.38 | 113.5±3.07 | 107.36±1.73 | 112.62±1.11 | 112.64±1.31 |
| WHR | 0.92±0.01 | 0.89±0.02 | 0.9±0.01 | 0.89±0.01 | 0.89±0.02 | 0.93±0.02 | 0.9±0.01 | 0.91±0.01 |
| AGR | 0.61±0.04 | 0.6±0.03 | 0.63±0.02 | 0.6±0.02 | 0.63±0.04 | 0.61±0.03 | 0.65±0.02 | 0.63±0.02 |
| **Indicators for defining MHO and MUO** | | | | | | | | |

| | | | | | | | | |
|---|---|---|---|---|---|---|---|---|
| SBP (mmHg) | 120.5±1.3 | 112.25±2.17 | 123.57±1.74 | 126.57±2.98 | 138.33±1.93 | 131.93±1.94 | 133.33±2.23 | 116.98±1.6 |
| DBP (mmHg) | 68.8±1.53 | 65.3±1.52 | 76.33±1.74 | 67.33±1.46 | 73.00±2.86 | 77.93±3.87 | 80.6±1.66 | 77.22±1.93 |
| FBG (mmol/L) | 4.88±0.08 | 4.62±0.08 | 5.04±0.10 | 4.72±0.09 | 4.98±0.05 | 4.93±0.20 | 5.33±0.12 | 4.94±0.12 |
| TG (mmol/L) | 0.91±0.06 | 1.02±0.06 | 1.4±0.12 | 1.13±0.06 | 1.15±0.12 | 1.25±0.08 | 1.98±0.18 | 1.64±0.11 |
| HDL-C (mmol/L) | 1.39±0.05 | 1.36±0.04 | 1.26±0.04 | 1.26±0.02 | 1.27±0.08 | 1.28±0.09 | 1.19±0.04 | 1.20±0.03 |
| **Other metabolic health-related indicators** | | | | | | | | |
| HOMA-IR | 3.02±0.26 | 2.30±0.21 | 4.29±0.79 | 2.36±0.22 | 3.48±0.46 | 2.48±0.50 | 5.57±0.59 | 3.33±0.62 |
| FINS (pmol/mL) | 96.75±8.02 | 77.88±6.63 | 127.68±18.84 | 79.10±7.92 | 109.4±14.44 | 78.23±15.8 | 162.14±15.35 | 99.53±13.79 |
| TC (mmol/L) | 4.54±0.15 | 4.09±0.14 | 4.66±0.13 | 4.08±0.12 | 5.09±0.36 | 4.37±0.19 | 4.74±0.15 | 4.16±0.15 |
| LDL-C (mmol/L) | 2.69±0.14 | 2.62±0.14 | 2.85±0.13 | 2.71±0.13 | 3.23±0.34 | 2.93±0.11 | 2.91±0.12 | 2.75±0.13 |
| HDL-C/TC | 0.31±0.02 | 0.34±0.02 | 0.28±0.01 | 0.32±0.01 | 0.26±0.02 | 0.29±0.01 | 0.26±0.01 | 0.30±0.01 |
| $VO_2max$ (ml/(kg·min) | 27.73±1.46 | 29.60±1.3 | 28.31±1.02 | 30.81±1.28 | 26.86±1.38 | 27.86±2.21 | 25.14±0.61 | 26.00±0.92 |

*Note: Values are expressed as Mean ± SE. Pre and Post represent measurements before and after the 8-week exercise intervention, respectively.*

**Table S5. Between-group comparison of changes (Δ) in amino acid metabolites between the MHO-R and MUO-C groups**

| Metabolite | ΔMHO-R | ΔMUO-C | *p*-value | FDR |
|---|---|---|---|---|
| 1-Methylhistidine | -0.015 | 0.074 | 0.341 | 0.989 |
| 2-Aminobutyric Acid | -1.825 | 0.999 | 0.495 | 0.989 |
| 3-Methylhistidine | 0.210 | 0.115 | 0.779 | 0.989 |
| Alanine | -0.952 | 0.488 | 0.583 | 0.989 |
| Aminoadipic Acid | -0.008 | 0.006 | 0.327 | 0.989 |
| Arginine | 9.661 | 9.073 | 0.602 | 0.989 |
| Asparagine | -0.025 | -0.010 | 0.883 | 0.989 |
| Carnosine | 0.014 | 0.015 | 0.820 | 0.989 |
| Citrulline | -0.360 | -0.340 | 0.883 | 0.989 |
| Creatinine | -0.176 | -0.004 | 0.192 | 0.989 |
| Cystine | 0.000 | 0.005 | 0.102 | 0.989 |
| Glutamate | -0.556 | -0.648 | 0.925 | 0.989 |
| Glutamine | -1.905 | -2.294 | 0.698 | 0.989 |
| Glycine | 0.686 | 0.795 | 0.659 | 0.989 |
| Histidine | 1.904 | 2.539 | 0.718 | 0.989 |
| Hydroxyproline | -0.100 | 0.025 | 0.640 | 0.989 |
| Isoleucine | -0.007 | 0.045 | 0.445 | 0.989 |
| Leucine | -0.029 | 0.469 | 0.547 | 0.989 |
| L-Homoserine | -0.330 | -0.428 | 0.968 | 0.989 |
| Lysine | 0.256 | 0.766 | 0.779 | 0.989 |
| Methionine | 0.001 | 0.004 | 0.478 | 0.989 |
| Ornithine | 0.226 | 0.357 | 0.583 | 0.989 |
| Phenylalanine | 0.026 | 0.162 | 0.165 | 0.989 |
| Proline | -0.124 | 0.048 | 0.134 | 0.989 |
| Sarcosine | -0.011 | 0.038 | 0.253 | 0.989 |
| Serine | 0.092 | 0.110 | 0.989 | 0.989 |
| Threonine | -0.127 | -0.167 | 0.904 | 0.989 |
| Tryptophan | -0.286 | 0.063 | 0.445 | 0.989 |
| Tyrosine | -0.065 | -0.047 | 0.529 | 0.989 |
| Valine | 0.395 | 0.670 | 0.799 | 0.989 |

*Note: Values are expressed as median changes (Post − Pre) within each group. The p-values refer to between-group comparisons of Δ values using the Mann–Whitney U test. False discovery rate (FDR) correction was performed for multiple comparisons. No metabolites remained statistically significant after FDR correction (FDR < 0.05).*

**Table S6. Within-group changes in amino acid metabolites after exercise intervention in the MHO-R and MUO-C groups (Pre vs Post)**

| Group | Metabolite | KEGGID | Class | FC | log2FC | *p*-value | FDR | Direction | Sig. |
|---|---|---|---|---|---|---|---|---|---|
| MHO-R | Arginine | C00062 | Amino acid | 1.824 | 0.867 | < 0.001 | 0.002 | ↑ | ** |
| MHO-R | Carnosine | C00386 | Amino acid and derivatives | 1.705 | 0.770 | < 0.001 | 0.002 | ↑ | ** |
| MHO-R | Histidine | C00135 | Amino acid | 1.584 | 0.664 | < 0.001 | 0.002 | ↑ | ** |
| MHO-R | Glycine | C00037 | Amino acid | 1.132 | 0.178 | 0.001 | 0.007 | ↑ | ** |
| MHO-R | Citrulline | C00327 | Amino acid and derivatives | 0.846 | -0.241 | 0.001 | 0.007 | ↓ | ** |
| MHO-R | Glutamine | C00064 | Amino acid | 0.928 | -0.108 | 0.003 | 0.013 | ↓ | * |
| MHO-R | L-Homoserine | C00263 | Amino acid and derivatives | 0.932 | -0.102 | 0.024 | 0.090 | ↓ | |
| MHO-R | Threonine | C00188 | Amino acid | 0.903 | -0.147 | 0.022 | 0.090 | ↓ | |
| MHO-R | Serine | C00065 | Amino acid | 1.052 | 0.073 | 0.029 | 0.097 | ↑ | |
| MHO-R | Valine | C00183 | Amino acid | 1.120 | 0.164 | 0.038 | 0.115 | ↑ | |
| MHO-R | Glutamate | C00025 | Amino acid | 0.837 | -0.257 | 0.059 | 0.148 | ↓ | |
| MHO-R | Phenylalanine | C00079 | Amino acid | 1.036 | 0.051 | 0.059 | 0.148 | ↑ | |
| MHO-R | Cystine | C00491 | Amino acid and derivatives | 1.053 | 0.075 | 0.065 | 0.149 | ↑ | |
| MHO-R | Methionine | C00073 | Amino acid | 1.489 | 0.575 | 0.089 | 0.192 | ↑ | |
| MHO-R | Sarcosine | C00213 | Amino acid and derivatives | 1.199 | 0.262 | 0.104 | 0.209 | ↑ | |
| MHO-R | Isoleucine | C00407 | Amino acid | 1.072 | 0.101 | 0.113 | 0.211 | ↑ | |
| MHO-R | Leucine | C00123 | Amino acid | 0.985 | -0.022 | 0.121 | 0.214 | ↓ | |
| MHO-R | 3-Methylhistidine | C01152 | Amino acid and derivatives | 1.309 | 0.388 | 0.131 | 0.218 | ↑ | |
| MHO-R | 1-Methylhistidine | C01152 | Amino acid and derivatives | 1.056 | 0.079 | 0.185 | 0.264 | ↑ | |
| MHO-R | Lysine | C00047 | Amino acid | 1.078 | 0.108 | 0.173 | 0.264 | ↑ | |
| MHO-R | Ornithine | C00077 | Amino acid and derivatives | 1.037 | 0.053 | 0.185 | 0.264 | ↑ | |
| MHO-R | Asparagine | C00152 | Amino acid | 0.975 | -0.037 | 0.271 | 0.369 | ↓ | |

| | | | | | | | | | |
|---|---|---|---|---|---|---|---|---|---|
| MHO-R | 2-Aminobutyric Acid | C02356 | Amino acid and derivatives | 1.065 | 0.091 | 0.563 | 0.704 | ↑ | |
| MHO-R | Creatinine | C00791 | Amino acid and derivatives | 0.951 | -0.072 | 0.563 | 0.704 | ↓ | |
| MHO-R | Proline | C00148 | Amino acid | 0.968 | -0.047 | 0.614 | 0.737 | ↓ | |
| MHO-R | Tyrosine | C00082 | Amino acid | 0.986 | -0.021 | 0.695 | 0.802 | ↓ | |
| MHO-R | Hydroxyproline | C01157 | Amino acid and derivatives | 0.968 | -0.047 | 0.779 | 0.866 | ↓ | |
| MHO-R | Aminoadipic Acid | C00956 | Amino acid and derivatives | 1.025 | 0.035 | 0.926 | 0.992 | ↑ | |
| MHO-R | Alanine | C00041 | Amino acid | 1.036 | 0.052 | 1.000 | 1.000 | ↑ | |
| MHO-R | Tryptophan | C00078 | Amino acid | 1.028 | 0.040 | 0.985 | 1.000 | ↑ | |
| MUO-C | Arginine | C00062 | Amino acid | 1.823 | 0.867 | $< 0.001$ | $9.6 \times 10^{-4}$ | ↑ | *** |
| MUO-C | Carnosine | C00386 | Amino acid and derivatives | 1.690 | 0.757 | $< 0.001$ | $9.6 \times 10^{-4}$ | ↑ | *** |
| MUO-C | Histidine | C00135 | Amino acid | 1.514 | 0.599 | $< 0.001$ | $9.6 \times 10^{-4}$ | ↑ | *** |
| MUO-C | Citrulline | C00327 | Amino acid and derivatives | 0.793 | -0.335 | 0.001 | 0.008 | ↓ | ** |
| MUO-C | Glycine | C00037 | Amino acid | 1.038 | 0.054 | 0.003 | 0.020 | ↑ | * |
| MUO-C | Glutamate | C00025 | Amino acid | 0.836 | -0.258 | 0.011 | 0.053 | ↓ | |
| MUO-C | 3-Methylhistidine | C01152 | Amino acid and derivatives | 1.451 | 0.537 | 0.014 | 0.054 | ↑ | |
| MUO-C | Serine | C00065 | Amino acid | 1.070 | 0.098 | 0.014 | 0.054 | ↑ | |
| MUO-C | Glutamine | C00064 | Amino acid | 1.007 | 0.010 | 0.035 | 0.105 | ↑ | |
| MUO-C | L-Homoserine | C00263 | Amino acid and derivatives | 0.915 | -0.128 | 0.035 | 0.105 | ↓ | |
| MUO-C | Threonine | C00188 | Amino acid | 0.856 | -0.224 | 0.046 | 0.125 | ↓ | |
| MUO-C | Tyrosine | C00082 | Amino acid | 0.945 | -0.081 | 0.065 | 0.162 | ↓ | |
| MUO-C | Proline | C00148 | Amino acid | 0.961 | -0.057 | 0.070 | 0.162 | ↓ | |
| MUO-C | Creatinine | C00791 | Amino acid and derivatives | 0.951 | -0.073 | 0.076 | 0.163 | ↓ | |
| MUO-C | Tryptophan | C00078 | Amino acid | 0.965 | -0.052 | 0.131 | 0.261 | ↓ | |
| MUO-C | Valine | C00183 | Amino acid | 1.117 | 0.160 | 0.151 | 0.282 | ↑ | |

| | | | | | | | | |
|---|---|---|---|---|---|---|---|---|
| MUO-C | Aminoadipic Acid | C00956 | Amino acid and derivatives | 0.880 | -0.185 | 0.162 | 0.285 | ↓ |
| MUO-C | Alanine | C00041 | Amino acid | 0.968 | -0.047 | 0.198 | 0.330 | ↓ |
| MUO-C | Asparagine | C00152 | Amino acid | 0.965 | -0.052 | 0.341 | 0.539 | ↓ |
| MUO-C | Lysine | C00047 | Amino acid | 1.044 | 0.063 | 0.360 | 0.541 | ↑ |
| MUO-C | Hydroxyproline | C01157 | Amino acid and derivatives | 1.060 | 0.084 | 0.514 | 0.734 | ↑ |
| MUO-C | Leucine | C00123 | Amino acid | 1.074 | 0.103 | 0.641 | 0.874 | ↑ |
| MUO-C | 2-Aminobutyric Acid | C02356 | Amino acid and derivatives | 0.951 | -0.073 | 0.723 | 0.904 | ↓ |
| MUO-C | Methionine | C00073 | Amino acid | 1.042 | 0.060 | 0.695 | 0.904 | ↑ |
| MUO-C | 1-Methylhistidine | C01152 | Amino acid and derivatives | 1.056 | 0.078 | 0.837 | 0.966 | ↑ |
| MUO-C | Ornithine | C00077 | Amino acid and derivatives | 1.055 | 0.077 | 0.808 | 0.966 | ↑ |
| MUO-C | Cystine | C00491 | Amino acid and derivatives | 0.970 | -0.044 | 0.985 | 1.000 | ↓ |
| MUO-C | Isoleucine | C00407 | Amino acid | 1.011 | 0.015 | 0.985 | 1.000 | ↑ |
| MUO-C | Phenylalanine | C00079 | Amino acid | 0.986 | -0.020 | 0.926 | 1.000 | ↓ |
| MUO-C | Sarcosine | C00213 | Amino acid and derivatives | 0.939 | -0.091 | 1.000 | 1.000 | ↓ |

*Note: This table presents amino acid metabolites identified by within-group comparisons (Post vs Pre) in the MHO-R and MUO-C groups following exercise intervention. KEGGID refers to the compound identifier in the KEGG database. FC indicates fold change (Post/Pre), and log2FC indicates log2-transformed fold change. Raw p-values were obtained using the Wilcoxon signed-rank test, and false discovery rate (FDR) correction was applied for multiple comparisons. Direction (↑/↓) indicates up- or down-regulation after intervention. Metabolites with FDR < 0.05 were considered statistically significant. Sig. indicates significance levels based on FDR-adjusted p-values (*FDR < 0.05, **FDR < 0.01, ***FDR < 0.001).*

**Table S7. Pathway enrichment analysis of exercise-responsive metabolites in the MHO-R and MUO-C groups (Pre vs Post).**

| Group | Pathway | Total | Hits | Expected | p-value | FDR | Impact | Enriched |
|---|---|---|---|---|---|---|---|---|
| MHO-R | Arginine biosynthesis | 14 | 3 | 0.053 | < 0.001 | $9.6 \times 10^{-4}$ | 0.319 | Yes |
| MHO-R | Histidine metabolism | 16 | 2 | 0.060 | 0.001 | 0.056 | 0.311 | |
| MHO-R | beta-Alanine metabolism | 21 | 2 | 0.079 | 0.002 | 0.064 | 0.056 | |
| MHO-R | Glyoxylate and dicarboxylate metabolism | 32 | 2 | 0.121 | 0.006 | 0.112 | 0.083 | |
| MHO-R | Nitrogen metabolism | 6 | 1 | 0.023 | 0.022 | 0.359 | 0.000 | |
| MHO-R | Pyrimidine metabolism | 39 | 1 | 0.147 | 0.138 | 0.852 | 0.000 | |
| MHO-R | Arginine and proline metabolism | 36 | 1 | 0.136 | 0.128 | 0.852 | 0.124 | |
| MHO-R | Glycine, serine and threonine metabolism | 33 | 1 | 0.124 | 0.118 | 0.852 | 0.260 | |
| MHO-R | Porphyrin metabolism | 31 | 1 | 0.117 | 0.111 | 0.852 | 0.000 | |
| MHO-R | Lipoic acid metabolism | 28 | 1 | 0.106 | 0.101 | 0.852 | 0.002 | |
| MHO-R | Glutathione metabolism | 28 | 1 | 0.106 | 0.101 | 0.852 | 0.089 | |
| MHO-R | Alanine, aspartate and glutamate metabolism | 28 | 1 | 0.106 | 0.101 | 0.852 | 0.114 | |
| MHO-R | One carbon pool by folate | 26 | 1 | 0.098 | 0.094 | 0.852 | 0.038 | |
| MHO-R | Primary bile acid biosynthesis | 46 | 1 | 0.173 | 0.162 | 0.923 | 0.008 | |
| MHO-R | Purine metabolism | 70 | 1 | 0.264 | 0.237 | 1.000 | 0.000 | |
| MUO-C | Histidine metabolism | 16 | 2 | 0.050 | 0.001 | 0.037 | 0.311 | Yes |
| MUO-C | Arginine biosynthesis | 14 | 2 | 0.044 | 0.001 | 0.037 | 0.319 | Yes |
| MUO-C | beta-Alanine metabolism | 21 | 2 | 0.066 | 0.002 | 0.043 | 0.056 | |
| MUO-C | Arginine and proline metabolism | 36 | 1 | 0.113 | 0.108 | 0.866 | 0.124 | |
| MUO-C | Glycine, serine and threonine metabolism | 33 | 1 | 0.104 | 0.100 | 0.866 | 0.260 | |
| MUO-C | Glyoxylate and dicarboxylate metabolism | 32 | 1 | 0.101 | 0.097 | 0.866 | 0.083 | |

| | | | | | | | |
|---|---|---|---|---|---|---|---|
| MUO-C | Porphyrin metabolism | 31 | 1 | 0.097 | 0.094 | 0.866 | 0.000 |
| MUO-C | Lipoic acid metabolism | 28 | 1 | 0.088 | 0.085 | 0.866 | 0.002 |
| MUO-C | Glutathione metabolism | 28 | 1 | 0.088 | 0.085 | 0.866 | 0.089 |
| MUO-C | One carbon pool by folate | 26 | 1 | 0.082 | 0.079 | 0.866 | 0.038 |
| MUO-C | Primary bile acid biosynthesis | 46 | 1 | 0.144 | 0.137 | 0.993 | 0.008 |

*Note: This table presents pathway enrichment results derived from within-group metabolite changes (Post vs Pre) in the MHO-R and MUO-C groups following exercise intervention. Total indicates the total number of metabolites within each pathway; Hits indicates the number of matched metabolites identified in the enrichment analysis; Expected indicates the expected number of hits by random chance; Raw p-values were calculated from pathway enrichment analysis, and false discovery rate (FDR) correction was applied for multiple comparisons. Impact indicates the pathway impact score. Enriched indicates pathways meeting the predefined enrichment criteria (FDR < 0.05, impact > 0.1, and Hits ≥ 2).*

**Table S8. Amino acid metabolite changes identified after combining the MHO-R and MUO-C groups (Pre vs Post).**

| Metabolite | KEGGID | Class | FC | log2FC | *p*-value | FDR | Direction | Sig. |
|---|---|---|---|---|---|---|---|---|
| Arginine | C00062 | Amino acid | 1.753 | 0.810 | <0.001 | $5.0 \times 10^{-7}$ | ↑ | *** |
| Carnosine | C00386 | Amino acid and derivatives | 1.710 | 0.774 | <0.001 | $5.0 \times 10^{-7}$ | ↑ | *** |
| Histidine | C00135 | Amino acid | 1.535 | 0.618 | <0.001 | $5.0 \times 10^{-7}$ | ↑ | *** |
| Citrulline | C00327 | Amino acid and derivatives | 0.820 | -0.287 | <0.001 | $2.7 \times 10^{-5}$ | ↓ | *** |
| Glycine | C00037 | Amino acid | 1.102 | 0.140 | <0.001 | $4.2 \times 10^{-5}$ | ↑ | *** |
| Glutamine | C00064 | Amino acid | 0.962 | -0.056 | <0.001 | 0.001 | ↓ | ** |
| Serine | C00065 | Amino acid | 1.065 | 0.092 | 0.001 | 0.005 | ↑ | ** |
| Glutamate | C00025 | Amino acid | 0.869 | -0.203 | 0.002 | 0.006 | ↓ | ** |
| L-Homoserine | C00263 | Amino acid and derivatives | 0.923 | -0.115 | 0.002 | 0.008 | ↓ | ** |
| Threonine | C00188 | Amino acid | 0.882 | -0.181 | 0.003 | 0.008 | ↓ | ** |
| 3-Methylhistidine | C01152 | Amino acid and derivatives | 1.393 | 0.478 | 0.008 | 0.021 | ↑ | * |
| Valine | C00183 | Amino acid | 1.085 | 0.118 | 0.011 | 0.027 | ↑ | * |
| Creatinine | C00791 | Amino acid and derivatives | 0.952 | -0.071 | 0.063 | 0.145 | ↓ | |
| Lysine | C00047 | Amino acid | 1.067 | 0.094 | 0.084 | 0.18 | ↑ | |
| Leucine | C00123 | Amino acid | 1.008 | 0.011 | 0.097 | 0.194 | ↑ | |
| Asparagine | C00152 | Amino acid | 0.974 | -0.038 | 0.121 | 0.213 | ↓ | |
| Phenylalanine | C00079 | Amino acid | 1.023 | 0.032 | 0.114 | 0.213 | ↑ | |
| Cystine | C00491 | Amino acid and derivatives | 1.033 | 0.047 | 0.152 | 0.228 | ↑ | |
| Methionine | C00073 | Amino acid | 1.156 | 0.210 | 0.145 | 0.228 | ↑ | |
| Tyrosine | C00082 | Amino acid | 0.925 | -0.113 | 0.137 | 0.228 | ↓ | |
| 1-Methylhistidine | C01152 | Amino acid and derivatives | 1.040 | 0.056 | 0.234 | 0.319 | ↑ | |
| Sarcosine | C00213 | Amino acid and derivatives | 0.966 | -0.049 | 0.224 | 0.319 | ↓ | |

| | | | | | | | |
|---|---|---|---|---|---|---|---|
| Ornithine | C00077 | Amino acid and derivatives | 1.056 | 0.078 | 0.285 | 0.372 | ↑ |
| Isoleucine | C00407 | Amino acid | 1.030 | 0.043 | 0.304 | 0.38 | ↑ |
| Tryptophan | C00078 | Amino acid | 0.996 | -0.006 | 0.357 | 0.429 | ↓ |
| Aminoadipic Acid | C00956 | Amino acid and derivatives | 0.979 | -0.030 | 0.379 | 0.437 | ↓ |
| Alanine | C00041 | Amino acid | 0.986 | -0.020 | 0.432 | 0.447 | ↓ |
| Hydroxyproline | C01157 | Amino acid and derivatives | 0.992 | -0.012 | 0.408 | 0.447 | ↓ |
| Proline | C00148 | Amino acid | 0.963 | -0.054 | 0.432 | 0.447 | ↓ |
| 2-Aminobutyric Acid | C02356 | Amino acid and derivatives | 1.053 | 0.075 | 0.995 | 0.995 | ↑ |

*Note: Differential amino acid metabolite changes were identified by comparing pre- and post-intervention data after combining the MHO-R and MUO-C groups, as no significant time × group interaction was observed. Metabolites with FDR < 0.05 were considered statistically significant. FC indicates fold change (Post/Pre), and log2FC indicates log2-transformed fold change. Direction (↑/↓) indicates up- or down-regulation after intervention. Sig. indicates significance levels based on FDR-adjusted p-values (*FDR < 0.05, **FDR < 0.01, ***FDR < 0.001).*

**Table S9. Pathway enrichment analysis after combining the MHO-R and MUO-C groups (Pre vs Post).**

| Pathway | Total | Hits | Expected | *p*-value | FDR | Impact | Enriched |
|---|---|---|---|---|---|---|---|
| Arginine biosynthesis | 14 | 4 | 0.097 | <0.001 | $8.6 \times 10^{-5}$ | 0.441 | Yes |
| Histidine metabolism | 16 | 4 | 0.111 | <0.001 | $8.6 \times 10^{-5}$ | 0.311 | Yes |
| Glyoxylate and dicarboxylate metabolism | 32 | 4 | 0.221 | <0.001 | 0.001 | 0.110 | Yes |
| Glycine, serine and threonine metabolism | 33 | 3 | 0.228 | 0.001 | 0.016 | 0.474 | Yes |
| Nitrogen metabolism | 6 | 2 | 0.041 | <0.001 | 0.013 | 0.000 | |
| Valine, leucine and isoleucine biosynthesis | 8 | 2 | 0.055 | 0.001 | 0.016 | 0.000 | |
| beta-Alanine metabolism | 21 | 2 | 0.145 | 0.008 | 0.097 | 0.056 | |
| One carbon pool by folate | 26 | 2 | 0.180 | 0.013 | 0.119 | 0.056 | |
| Glutathione metabolism | 28 | 2 | 0.193 | 0.015 | 0.119 | 0.108 | |
| Alanine, aspartate and glutamate metabolism | 28 | 2 | 0.193 | 0.015 | 0.119 | 0.311 | |
| Porphyrin metabolism | 31 | 2 | 0.214 | 0.018 | 0.132 | 0.000 | |
| Arginine and proline metabolism | 36 | 2 | 0.249 | 0.024 | 0.160 | 0.124 | |
| D-Amino acid metabolism | 15 | 1 | 0.104 | 0.099 | 0.567 | 0.000 | |
| Butanoate metabolism | 15 | 1 | 0.104 | 0.099 | 0.567 | 0.000 | |
| Pantothenate and CoA biosynthesis | 20 | 1 | 0.138 | 0.130 | 0.694 | 0.000 | |
| Lipoic acid metabolism | 28 | 1 | 0.193 | 0.178 | 0.889 | 0.002 | |
| Sphingolipid metabolism | 32 | 1 | 0.221 | 0.201 | 0.917 | 0.000 | |
| Cysteine and methionine metabolism | 33 | 1 | 0.228 | 0.206 | 0.917 | 0.022 | |
| Pyrimidine metabolism | 39 | 1 | 0.269 | 0.239 | 0.979 | 0.000 | |
| Valine, leucine and isoleucine degradation | 40 | 1 | 0.276 | 0.245 | 0.979 | 0.000 | |
| Primary bile acid biosynthesis | 46 | 1 | 0.318 | 0.276 | 1.000 | 0.008 | |
| Purine metabolism | 70 | 1 | 0.484 | 0.391 | 1.000 | 0.000 | |

*Note: Pathway enrichment analysis was performed based on differential metabolite changes (Pre vs Post) identified after combining the MHO-R and MUO-C groups. Pathways meeting the predefined enrichment criteria (FDR < 0.05, impact > 0.1, and Hits ≥ 2) were considered significantly enriched. Total indicates the total number of metabolites within each pathway; Hits indicates the number of matched metabolites identified in the enrichment analysis; Expected indicates the expected number of hits by random chance; Impact indicates the pathway impact score. Enriched indicates pathways meeting the predefined enrichment criteria.*
